# Assessing Barriers and Facilitators for Implementing Clinical Practice Guidelines in Middle Eastern and North African Region: Delphi Study

**DOI:** 10.3390/jcm12155113

**Published:** 2023-08-03

**Authors:** Saja H. Almazrou, Hajar Almoajil, Sara Alghamdi, Ghadeer Althenyan, Abdulhadi Alqahtani, Yasser Sami Amer

**Affiliations:** 1 College of Pharmacy, King Saud University, Riyadh 14511, Saudi Arabia; sarabalghamdi1@gmail.com (S.A.); ghadeerft@outlook.com (G.A.); 2 Physical Therapy Department, College of Applied Medical Sciences, Imam Abdulrahamn bin Faisal University, Dammam 34212, Saudi Arabia; hajar.almoajil@gmail.com; 3 Clinical Research Department, Research Center, King Fahad Medical City, Riyadh 11525, Saudi Arabia; amohalqahtani@kfmc.med.sa; 4 Pediatrics Department, King Saud University Medical City, Riyadh 11362, Saudi Arabia; yassersamiamer@gmail.com; 5 Clinical Practice Guidelines & Quality Research Unit, Quality Management Department, King Saud University Medical City, Riyadh 11362, Saudi Arabia; 6 Research Chair for Evidence-Based Health Care and Knowledge Translation, Family and Community Medicine Department, College of Medicine, King Saud University, Riyadh 11421, Saudi Arabia; 7 Alexandria Center for Evidence-Based Clinical Practice Guidelines, Alexandria University, Alexandria 5424041, Egypt; 8 Guidelines International Network, Perth PH16 5BU, UK

**Keywords:** clinical practice guidelines, implementation

## Abstract

Background: Clinical practice guidelines (CPGs) improve clinical decision making and patient outcomes, but CPG implementation is poor. The success of CPGs is influenced by several factors related to barriers and facilitators. For this reason, it can be extremely useful to explore key barriers and facilitators of CPG implementation in the Middle East and North Africa (MENA). Methods: A three-round Delphi study was performed using the input of 30 experts involved in the clinical practice guidelines. In the first two rounds, participants were asked to score each statement relevant to barriers or facilitators for CPG implementation on a five-point Likert scale. These statements were identified from existing systematic reviews and expert input. In round three, participants ranked the most important barriers and facilitators identified from rounds one and two. A descriptive analysis was conducted on the barrier and facilitators statements using frequencies, percentages, and medians to summarize the variables collected. Results: We identified 10 unique barriers and 13 unique facilitators to CPG implementation within the MENA region. The two highest-ranked barriers related to communications and available research and skills. The most important facilitator was the availability of training courses for healthcare professionals. Conclusions: Key barriers and facilitators to the implementation of clinical practice guidelines seem to exist in professional, organizational, and external contexts, which should all be taken into account in order to increase implementation success within MENA region. The results of this study are useful in the design of future implementation strategies aimed at overcoming the barriers and leveraging the facilitators.

## 1. Introduction

The National Academy of Medicine, formerly called the Institute of Medicine until 2015, defined Clinical Practice Guidelines (CPGs) as “systematically developed statements to assist practitioner and patient decisions about appropriate healthcare for specific clinical circumstances”. They contain recommendations based on evidence from rigorous systematic reviews synthesized from published medical literature [1].

Over the last few decades, CPGs have become part of everyday practice; they are used to improve the quality of care [2]. CPGs are based on a critical appraisal of scientific evidence clarifying which interventions are of proven benefit and documenting the quality of the supporting data. Potentially benefiting patients, CPGs improve health outcomes by promoting proven interventions and discouraging ineffective ones, thus reducing morbidity and mortality, improving quality of life, and providing standardization of care [3]. 

CPGs also provide a consumer version that helps patients make informed choices about their health and consider their personal needs and preferences in selecting the best option [4]. This may influence public policy and public health because it calls attention to under-recognized health problems, clinical services, and preventive interventions, as well as neglected patient populations and high-risk groups that come to light when releasing new guidelines.

Benefits for healthcare professionals (HCPs) include improving the quality of clinical decisions by offering an evidence-based intervention that reassures HCPs about the appropriateness of their treatment and improves the consistency of care [5].

In 2020, a systematic review of barriers to and facilitators of adherence to CPGs in the MENA region [6] included studies from Saudi Arabia, Palestine, Egypt, Jordan, Iran, United Arab Emirates, and Sudan. The results showed that the most reported barriers to implementing CPG were environmental factors, such as a lack of protocols and processes for dissemination and implementation, lack of resources (staff, equipment, and beds), lack of clinical audit and feedback, and lack of training. Clinician-related factors included a lack of awareness of the existence of guidelines, a lack of familiarity with CPG recommendations, and disagreement with the guidance of CPGs. This review also reports that facilitators of adhering to guidelines include disseminating and advertising guideline materials, education and training on the guidelines, regulatory and financial incentives, and support from institutions. 

This systematic review reports 26 barriers and facilitators for implementing CPGs in the MENA region. This high number of factors may limit CPG implementation. Thus, our aim is to determine the most important barriers and facilitators using the Delphi method, which will eventually guide decision makers to implement specific strategies targeting the most important factors, in turn improving the healthcare system and its delivery and enhancing patient outcomes.

## 2. Materials and Methods

### 2.1. Study Design

The barriers and facilitators were prioritized (rated) by participants in three rounds of web-based Delphi surveys created in SurveyMonkey [7,8]. Using an online survey is advantageous to increase the feasibility of a wider population within MENA region sampling. 

We consulted studies assessing barriers to and facilitators for implementing CPGs to compile two lists: one for barriers and one for facilitators [2,6,9]. This process yielded a total of (22) barriers and (11) facilitators.

### 2.2. Study Participants

This study included researchers and scholars who were either involved in developing guidelines or publishing papers assessing barriers to and facilitators of implementing CPGs in the MENA region. 

We identified potential participants by referring to the reference lists of two systematic reviews conducted on CPGs in the MENA region [6,10]. In addition, we searched PubMed, Google, LinkedIn, and ResearchGate to identify all authors’ contact information. Finally, participants identified from reviews and the Guideline International Network Conference were asked to send the survey to other researchers in the field so that these individuals could also participate in this study.

### 2.3. Pilot Test

The survey was pre-tested through two phases. In the first phase, the pilot survey was sent to three different researchers who had published research in clinical practice. They were asked to review the content and the appropriateness of the survey language and length, as well as if there were any additional barriers and facilitators that needed to be added to the list. This phase yielded (4) barriers and (4) facilitators. 

In the second phase, they were provided with the final web-based survey to assess the ease of navigation and the changes made following the previous phase. Then, the survey was amended accordingly.

### 2.4. Consent

Eligible participants were sent an email that contained the information sheet and an invitation to participate in this study. The email contained a link to the electronic survey. When participants clicked on the link, their web browser opened the first page of the survey, which repeated the same study information provided in the email. Participants had to check a box that stated, “I have read the information sheet, and I agree to participate in this study survey, which will utilize the information for scientific research purposes”, before proceeding to the next page. Once eligible participants agreed to participate and consented, they were asked for an email address to register within the survey to be contacted in the future for a reminder and the second-round survey invitation.

### 2.5. First Round

The first part of the survey was a series of statements on the barriers to and facilitators of implementing CPGs in the MENA region to elicit participants’ perceived importance of those factors. Respondents were asked to rate each statement using a 5-point scale, from 1 (not at all important) to 5 (very important). There was an optional free-text field to allow participants to express their opinions in more detail on each statement and to suggest any additional statements. This was followed by participants’ demographics, which included age, gender, qualification, nationality, country of practice, years of experience, and expertise with CPGs. The round was open for 4 weeks, and reminder emails were sent to non-responders after two weeks to increase the response rate. 

The responses from the first survey were analyzed and used to create the second-round survey. Items scored between 4 and 5 (important) by ≥70% were considered and excluded from the second round. Items scored between 1 and 2 (not important) by ≥70% were excluded from the second round. Only items scored 3 (neutral) were included in the second-round survey for re-rating. Additional items suggested by participants in the free-text field were reviewed and carried forward to the second round.

### 2.6. Second Round

Participants were asked to re-score statements from the previous survey that did not reach our pre-defined criteria in the previous round. Similar to round one, respondents were asked to rate the statements on a 5-point Likert scale, and the round was open for 4 weeks, with reminder emails sent to non-responders after two weeks. Participants were also asked to write any additional items in the free-text field.

### 2.7. Ranking Round

All participants who had completed the first- and/or second-round survey were invited to rank the statements by their importance, with 1 as the most important statement, that reached the pre-defined criteria from the previous rounds. This round was open for 4 weeks.

### 2.8. Statistical Analysis

Descriptive statistics were conducted using Microsoft Excel (Microsoft Corporation, 2016) [11] to summarize the distribution of scores (median) and calculate the spread and agreement for each Delphi survey item interquartile range (IQR) and demonstrate the most and least perceived barriers and facilitators (percentage).

## 3. Results

### 3.1. Expert Panel (Participants) 

A total of 198 participants were identified by their publications, including the corresponding authors and all co-authors. In addition, 19 participants were identified from the Guideline International Network Conference. Out of 217 invited, 32 (13%) responded to the invitation. Two participants did not complete demographic information. Sixteen of the 30 participated in both rounds. The majority of study participants had more than 15 years of experience and held a doctorate degree. Table 1 summarizes the participants’ characteristics.

### 3.2. First-Round Results

Twenty-five participated in this round. A total of 8 out of 41 statements reached consensus as being of importance by participants. Out of the eight statements, two were barriers, and six were facilitators, consensus ranged from 72% to 80% and 70% to 78%, respectively. No statement reached a consensus of no importance (i.e., consensus out) by participants.

### 3.3. Additional Factors

An additional nine statements were suggested by participants. Out of these, six were already included in the existing statements, and three were new statements included in the second-round survey for rating: a lack of regular review and update of existing CPGs, clinical and quality champions supporting the implementation of these specific CPGs, and networking with existing organizational projects (e.g., accreditation, scientific production, patient safety initiatives, etc.). 

In addition, one participant suggested re-wording the statement “Lack of training in CPGs implementation” to become “Lack of training in CPG evaluation, development/adaptation, and/or implementation”. Based on their feedback, we added two additional statements: lack of training in CPG evaluation and lack of training in CPG adaption. By the end of this round, a total of five new statements were added to the second-round survey. 

### 3.4. Second-Round Results 

This round included 21 participants. A total of 15 out of 38 statements reached consensus as being of importance by participants. Out of the 15 statements, 8 were barriers, and 7 were facilitators; consensus ranged from 71% to 90% and 71% to 71%, respectively. No statement reached a consensus of no importance (i.e., consensus out) by participants. 

### 3.5. Additional Factors

New factors suggested by participants included pharma influence on physicians’ practice via different types of incentives, a lack of collaboration between Arab countries, having CPGs (especially national CPGs) embedded into training and educational curricula with the Saudi Commission for Health Specialties (SCFHS) for medicine, nursing, pharmacy, and other healthcare training and education and certification, and emphasizing the role of the media in reaching particularly young physicians. 

Table 2 and Table 3 summarize the percentage of first- and second-round participants rating each barrier and facilitator statement as 1–2, 3, and 4–5 on the 5-point scale, respectively.

### 3.6. Ranking Round Results 

By the end of the first and second rounds, a total of 10 barriers and 13 facilitators reached a consensus as being of importance. All participants who had completed the first and/or second round surveys were invited to rank the statements (1 = the most important). A total of 13 participants completed this round. The top-rated barriers were healthcare practitioner (HCP) preferences for experience over CPGs as the top barrier. This was followed by a lack of policymaker support, then a lack of motivation. The top-rated facilitators were increasing the awareness about CPG, followed by enhancing the accessibility to CPG, and finally, customizing CPG to the local context.

Table 4 and Table 5 summarize the overall ranking for barrier and facilitator statements, respectively. 

## 4. Discussion

This cross-sectional survey used the Delphi Method to identify the potential facilitators of and barriers to implementing CPGs. The authors support the increasing need and trend of published studies aiming to identify tools, strategies, and interventions to enhance CPG implementation [12,13,14,15,16,17,18,19,20]. Identifying the facilitators of and barriers to implementing the recommendations of a CPG and suggesting strategies and interventions to enhance these facilitators and overcome these barriers are considered vital components of high-quality CPGs. The Applicability Domain (Domain 5) of the Appraisal of Guidelines for Research & Evaluation II (AGREE II) Instrument, the gold standard for appraising the quality of CPGs, includes these facilitators and barriers as well [21]. 

Similar findings were observed in studies conducted to explore the potential challenges for implementing CPGs at the level of a specific health topic or healthcare service (e.g., breast cancer, diabetes mellitus, asthma, care of amputee patients, neonatal resuscitation, medication prescription, and pediatric gastroesophageal reflux disease), a specific healthcare provider’s perspective (e.g., physicians), a specific country (e.g., Australia, China, Lebanon, and South Africa), or a specific geographical region (e.g., thyroid cancer in the Asia-Pacific Region [22,23,24,25,26,27,28,29,30,31,32,33,34]).

A recent systematic review that aimed to identify barriers and enablers of implementing clinical practice guidelines in primary care suggests that policy-driven strategies targeting integrated models and multidisciplinary teams as well as increasing the involvement of patients and healthcare providers are essential to improve the implementation process [35]. 

Published evidence identified barriers not specified verbatim in our survey due to the differences in each study’s scope (e.g., nationwide vs. regional or topic-specific vs. general). These included financial restrictions, a lack of access to management resources and therapies, interdisciplinary training and education, geographical location within a country related to the healthcare context, accessibility of healthcare services, and the national healthcare system that supports the implementation of CPGs [24,26,32]. Nevertheless, our survey has captured a relevant item: the lack of policymaker support.

Peters et al. (2020) and Fu et al. (2022) highlighted the importance and impact of multidisciplinary teams on successful evidence-based CPG implementation [36,37]. Two studies in the MENA region identified similar CPG implementation barriers at an organizational level (Saudi Arabia) and a national level (Lebanon [13,34]).

Several organizations have research groups that study CPG implementation and its tools, strategies, and challenges, like the GIN Implementation Working Group and the Joanna Briggs Institute [18,38,39,40].

Furthermore, the World Health Organization (WHO) and its eastern Mediterranean regional office identified and recommended CPG implementation solutions in several of its publications and reports relevant to the region [41,42]. 

Mazza and colleagues proposed a taxonomy to classify CPG implementation strategies that address similar barriers, including quality improvement and performance management systems, information and communication technology applications, and dissemination strategies [43].

To the best of our knowledge, this is the first cross-sectional study and Delphi survey to explore the facilitators and barriers to CPG implementation in the Middle East and North Africa (MENA). One limitation of this study is that the survey respondents lacked representation of patients or people to bring to the discussion their preferences, values, and preferences as a central pillar of evidence-based healthcare. Recruiting patients was not feasible because the scope of our study was CPG implementation in general rather than CPG implementation of a specific health topic, unlike other studies [27]. 

In addition, clinicians’ views on the implementation and adherence to clinical practice guidelines were not included. However, future studies should include the clinicians’ views to gain a deeper insight into this issue. 

The authors believe that the primary set of identified 13 facilitators and 10 barriers should be taken into consideration and addressed by a multidisciplinary collaborative partnership between policymakers, clinical content or topic experts (all relevant healthcare providers), quality improvement and CPG implementation experts, and patient and public representatives [44]. In the case of real-world national CPG implementation, this partnership should include a representation of different healthcare sectors (primarily academic medical centers) at a national level and regulatory and accreditation bodies. 

Long-term sustainability over time beyond the initial implementation of CPGs should be the ultimate goal, and the selection of the appropriate sustainability model (e.g., process-staff-organization) may differ between healthcare organizations, sectors, departments, and systems in each country, which is a recommendation for future research in our region [45,46].

CPG experts and researchers in the region hold great expectations for the GIN Arab Regional Community to collaborate and work on these regional research needs. Our study’s findings highlight the need for additional research to assess the facilitators and barriers to guideline implementation in specific countries of the MENA region, as well as the development of new evidence-based clinical practice guidelines that account for these identified regional factors.

## 5. Conclusions

Healthcare professionals and policymakers in MENA region believe that barriers and facilitators to CPGs are important factors in supporting implementation and maintenance. Key barriers and facilitators to the implementation of CPGs seem to endure in professional, organizational, and external contexts. All these levels should be taken into account in order to increase implementation success within MENA region. The results of this study are useful in the design of future implementation strategies aimed at overcoming the barriers and leveraging the facilitators.

## Figures and Tables

**Table 1:** Participants characteristics.

Participants Characteristics (n = 30)	% (N)
Age	
<30	0%
30–40	6% (2)
40–50	31% (10)
>50	56% (18)
Gender	
Male	59% (19)
Female	34% (11)
Years of experience	
<10 years	6% (2)
10–15 years	9% (3)
>15 years	78% (25)
Qualification	
Doctorate	81% (26)
Master’s	9% (3)
Undergraduate	3% (1)
Profession	
Physician	50% (16)
Pharmacist	9% (3)
Nurse	6% (2)
Healthcare policymaker	6% (2)
Quality manager/coordinator	3% (1)
Other	19% (6)
Affiliation	
Governmental or military hospitals	19% (6)
Private hospitals	6% (2)
Teaching hospitals	28% (9)
Public health institution	3% (1)
Academic	25% (8)
Ministry of Health	3% (1)
Other	9% (3)
Expertise with CPGs (multiple choice)	
Guideline development group member	63% (20)
Researcher in evidence-based medicine	47% (15)
Healthcare practitioner	44% (14)
Administrator	6% (2)
Quality coordinator	16% (5)
Other	13% (4)
Nationality	
Saudi Arabia	22% (7)
Iran	3% (1)
Kuwait	3% (1)
Jordan	13% (4)
Lebanon	13% (4)
Egypt	16% (5)
Sudan	6% (2)
Palestine	6% (2)
Tunisia	3% (1)
Yemen	3% (1)
Main Country of practice and/or research within MENA region (multiple choice)	
Saudi Arabia	47% (15)
Iran	3% (1)
Kuwait	3% (1)
Jordan	3% (1)
Lebanon	9% (3)
United Arab Emirates	3% (1)
Egypt	9% (3)
Sudan	3% (1)
Palestine	3% (1)
Tunisia	3% (1)
Other	9% (3)

**Table 2:** Descriptive analysis of barriers statements included in the first and second rounds.

Potential Barriers to Implementing Clinical Practice Guidelines in the MENA Region												
Item	First-Round Results						Second-Round Results					
	1–2	3	4–5	M	IQR	C	1–2	3	4–5	M	IQR	C
Lack of awareness of the existence of CPGs.	0%	44%	56%	4	2	N	0%	38%	62%	4	2	N
Lack of familiarity with CPG recommendations.	0%	36%	64%	4	2	N	5%	29%	67%	4	2	N
Lack of agreement with CPG recommendations.	16%	32%	52%	4	2	N	24%	24%	52%	4	1	N
Lack of certainty of the positive impact of CPGs on patient health outcomes.	20%	36%	40%	3	1	N	5%	43%	52%	4	1	N
Healthcare practitioners (HCPs) preference for experience over CPGs.	4%	20%	72%	4	1.25	Y						
Lack of effective communication between the healthcare team.	4%	36%	52%	4	2	N	0%	0%	90%	5	0.5	Y
Lack of effective research and self-learning skills.	8%	32%	56%	4.5	2	N	0%	10%	86%	4	1	Y
Lack of motivation.	20%	20%	56%	4	2	N	10%	14%	76%	4	1	Y
CPG complexity or lack of clarity.	36%	16%	48%	3	2	N	10%	24%	67%	4	2	N
Lack of CPGs’ trustworthiness (i.e., evidence quality, content, and developers).	16%	28%	56%	4	2	N	24%	29%	48%	3	1	N
CPGs limit treatment options.	52%	20%	24%	2	1.5	N	48%	29%	19%	2.5	1	N
CPGs limit flexibility or individual approach.	52%	16%	32%	2	2	N	52%	19%	24%	2	1.25	N
Lack of diversity in CPGs development that limits its application (i.e., lack of population diversity).	48%	8%	44%	3	2	N	33%	29%	33%	3	2	N
Lack of CPG customization to practice settings, culture, and patient groups.	28%	28%	44%	3	2	N	0%	24%	71%	4	1.25	Y
Lack of evidence from local settings.	16%	20%	64%	4	2	N	14%	10%	71%	4	1.25	Y
Lack of evidence-based culture and education.	12%	32%	56%	4	2	N	5%	14%	67%	4.5	1	N
Lack of protocols and processes for CPG dissemination.	12%	20%	64%	4	2	N	5%	14%	67%	5	1	N
Lack of time.	48%	24%	28%	3	2	N	14%	38%	43%	3	1	N
Lack of training in CPG implementation.	12%	28%	56%	4	2	N	0%	10%	81%	5	1	Y
Lack of resources (i.e., medicine, equipment, and tests recommended by CPGs).	20%	24%	52%	4	2	N	24%	24%	52%	4	1	N
Lack of insurance covering CPG recommendations.	16%	8%	52%	4	2	N	14%	33%	43%	3	1.5	N
Lack of high-quality enforced national guidelines.	4%	32%	56%	4	2	N	0%	10%	67%	5	1	N
Lack of policymaker support.	4%	16%	80%	5	1	Y						
Lack of regulation and supervision.	8.%	24%	68%	4	2	N	0%	29%	57%	4	2	N
Lack of clinical audit and feedback.	0%	28%	68%	4.5	2	N	0%	19%	71%	4	1	Y
Lack of financial incentives.	20%	24%	56%	4	1	N	14%	29%	52%	4	1	N
Lack of training in CPG evaluations.							5%	14%	67%	5	1	N
Lack of training in CPG adaptations *.							10%	5%	71%	5	1	Y
Lack of regular review and update of existing CPGs *.							0%	29%	67%	4	1.25	N

M = Mean, IQR = Interquartile range, C = Consensus, N = No, Y = Yes * Added in the second round.

**Table 3:** Descriptive analysis of facilitator statements included in the first and second rounds.

Potential Facilitators of Implementing Clinical Practice Guidelines in the MENA Region												
Item	First-Round Results						Second-Round Results					
	1–2	3	4–5	m	IQR	C	1–2	3	4–5	M	IQR	C
If CPGs are recommended by a colleague.	22%	26%	52%	4	1	N	10%	38%	52%	4	1	N
If HCPs are aware and educated about CPGs.	0%	17%	78%	5	1	Y						
If CPGs are in line with the majority of clinical scenarios seen in daily practice.	0%	17%	78%	4	1	Y						
If CPGs are flexible, allowing HCPs to make their own conclusion.	4%	22%	70%	4	1.75	Y						
If CPGs weigh patient preferences.	13%	13%	65%	4	1	N	14%	24%	62%	4	1	N
If CPGs could be accessed during patient care in a timely and easy manner.	9%	22%	70%	5	2	Y						
If CPGs are customized to local settings and needs.	4%	17%	78%	5	1	Y						
If CPGs are available in plain language summary directed to the patients.	13%	39%	48%	3	2	N	10%	19%	71%	4	2	Y
If CPG materials are advertised and disseminated.	4%	35%	61%	4	2	N	10%	10%	71%	4	1	Y
If CPGs are obligated by the institution/department head.	4%	35%	61%	4	2	N	0%	10%	81%	5	0	Y
If there is liability litigation associated with malpractice.	9%	30%	52%	4	2	N	0%	24%	67%	4	1.5	N
If there is organizational support to implement CPGs.	0%	26%	74%	5	1.5	Y						
If CPG training courses are available to HCPs.	9%	35%	52%	4	2	N	5%	5%	81%	5	1	Y
If a consultation team is available to answer questions about CPGs.	13%	30%	57%	4	2	N	5%	19%	71%	5	1.25	Y
If there are financial incentives.	9%	22%	65%	4	1.7	N	19%	14%	62%	4	2	N
If clinical and quality champions support the implementation of specific CPGs.							0%	24%	71%	4.5	1.25	Y
Networking with existing organizational projects (e.g., accreditation, scientific production, patient safety initiatives, etc.) *							5%	14%	76%	4.5	1	Y

M = Mean, IQR = Interquartile range, C = Consensus, N = No, Y = Yes. * Added in the second round.

**Table 4:** Summary of the overall ranking for barrier statements.

Ranking	Barriers to Implementing CPGs in MENA Region (n = 13)
1	Healthcare practitioner (HCP) preference for experience over CPGs.
2	Lack of policymaker support.
3	Lack of motivation.
4	Lack of training in CPG implementation.
5	Lack of CPG customization to practice settings, culture, and patient groups.
6	Lack of clinical audit and feedback.
7	Lack of evidence from local settings.
8	Lack of effective communication in the healthcare team.
9	Lack of effective research and self-learning skills.
10	Lack of training in CPG adaptations.

**Table 5:** Summary of the overall ranking for facilitator statements.

Ranking	Facilitators for Implementing CPGs in MENA Region (n = 13)
1	If HCPs are aware and educated about CPGs.
2	If CPGs could be accessed during patient care in a timely and easy manner.
3	If CPGs are customized to local settings and needs.
4	If CPGs are in line with the majority of clinical scenarios seen in daily practice.
5	If there is organizational support to implement CPGs.
6	If CPGs are obligated by the institution/department head.
7	If CPGs are flexible, allowing HCPs to make their own conclusions.
8	Networking with existing organizational projects (e.g., accreditation, scientific production, patient safety initiatives, etc.).
9	If clinical and quality champions support the implementation of specific CPGs.
10	If CPG materials are advertised and disseminated.
11	If CPG training courses are available to HCPs.
12	If CPGs are available in plain language summary directed to the patients.
13	If a consultation team is available to answer questions about the CPGs.

## Data Availability

Study data is available upon request.
